# Editorial: Hard facts or half-truths? The social and economic sustainability impact of flexible work practices in organizations

**DOI:** 10.3389/fpsyg.2022.1114627

**Published:** 2023-01-25

**Authors:** Emmanuel Aboagye, Beate Muschalla, Timo Lorenz, Aikaterini Grimani

**Affiliations:** ^1^Unit of Intervention and Implementation Research for Worker Health, Institute of Environmental Medicine, Karolinska Institute, Stockholm, Sweden; ^2^Department of Psychotherapy and Diagnostics, Technische Universität Braunschweig, Brunswick, Germany; ^3^Department of Psychology, Medical School Berlin, Berlin, Germany; ^4^Behavioural Science Group, Warwick Business School, University of Warwick, Coventry, United Kingdom

**Keywords:** flexible work arrangement practices, corporate leaders, organizational outcomes, idiosyncratic deals, social and economic sustainability, telework attitudes, performance

## Introduction

Existing literature indicates that flexible work seems to be an ambivalent experience (Putnam et al., 2013). Although it can enable employees regarding their work choices resulting in positive outcomes (i.e., work autonomy, job satisfaction, a better work/family life balance) (Tavares, 2017; Bhattacharya and Ray, 2021), the way that organizations implement work–life initiatives sometimes increases employees' work hours resulting in negative outcomes (i.e., job stress, loss of leisure time, reduced compensation, or a lack of predictable workweek, lack of support and isolation) (Kelliher and Anderson, 2009; Ray and Pana-Cryan, 2021; Gerich, 2022; Lunde et al., 2022). Due to our limited understanding, an increasing number of organizations are debating whether to continue with remote work or other hybrid teleworking forms (Golden, 2001; Neeley, 2021).

The present Research Topic addresses the following research questions:

- What is the relationship between flexible work practices and employee and organizational-level outcomes such as employee and leader behavior, health and work performance, and overall productivity?
- What types of flexible work practices might be more socially and economically sustainable (i.e., considering occupational categories, individual workers, and corporate leaders)? Studies on the perspectives of corporate leaders on the implications of these practices on their leadership capacity, managing employee behavior, and providing support to employees and peer leaders during the transition were also relevant to the topic.

## The current issue

Weber et al. investigated whether post-pandemic teleworking inclinations are influenced by teleworking conditions and perceived changes in productivity during the pandemic. A sample of 184 teleworkers across three primary countries participated in this cross-sectional study. Most employees wanted to telework more post-pandemic than before COVID-19. Although job demand was strongly associated with post-pandemic teleworking inclinations, other teleworking conditions such as job change, job control, home office adequacy, childcare had no observed effect on such tendencies. A relationship between work privacy fit and post-pandemic teleworking inclinations was also present, mediated by productivity perceptions. The study contributes to a more nuanced understanding of how contextual teleworking conditions may influence future teleworking inclinations, as well as to the identification of characteristics of employees who may not benefit from home-based teleworking post-pandemic due to their working conditions.

During the first year of the COVID-19 pandemic, Ipsen et al. investigated managers' experiences with distance management, their perceptions of organizational support, and the impact it had on their job satisfaction. Line, middle, and top managers (*n* = 1,016) participating in this study were recruited from Danish workplaces. One year after the COVID-19 pandemic began, most distance managers found their jobs to be more demanding and required them to work longer hours. Managers perceived that their own employees and manager peers provided the most support, while administrative support was largely lacking. Consequently, it seems that improving support from top management and in-house support functions would help maintain or increase managers' job satisfaction if an organization aims to offer hybrid work forms. The study adds to our understanding of the important role of perceived support on distance manager's job satisfaction because most managers believe they can adapt to the new role of distance management if necessary post-pandemic.

Idiosyncratic deals are sort of custom-made work arrangements negotiated between employees and employers to their benefit. This human resource management practices are critical in attracting, retaining, and motivating employees to foster innovation. Fan et al. investigated the relationship between work arrangements such as idiosyncratic deals and innovation at the team level. The study also examined the mediating role of team knowledge sharing, and the moderating roles of team transactive memory systems and team cognitive flexibility on innovation. Using a cross-sectional survey of 80 teams (*n* = 406 employees) from six enterprises in Shanghai and Hangzhou, the study found that higher idiosyncratic deals are associated with higher breakthrough innovation at the team level. This finding is mediated by higher team knowledge sharing which is positively moderated by the team's transactive memory systems and team cognitive flexibility. Moreover, the mediating effect of team knowledge sharing is stronger when high team transactive memory systems and high team cognitive flexibility are combined. The findings contribute to a more comprehensive view of this non-standard work arrangement where the influence of idiosyncratic deals at the team level seem to promote the sustainable growth of team innovation breakthroughs.

Mutiganda et al. conducted a systematic review investigating the relationship between telework and organizational economic outcomes such as self-reported employee performance, organizational performance, actual employee turnover rates, or intentions. Forty-three studies of moderate to high-quality were included with some addressing multiple outcomes. The findings were that: 1. Teleworking employees have higher perceived performance than those who work on the employer's premises; 2. Telework is associated with increased organizational performance, particularly in homogeneous samples with unique work tasks; and 3. Voluntary telework appears to lower both actual turnover rates and turnover intentions. This in-depth synthesis of the literature could help decision-makers better understand various teleworking arrangements and their economic implications for various organizational outcomes. The study also identifies some of the most likely elements associated with telework-related organizational economic losses and suggests future research into organizational telework practices.

## Conclusion

Many high-profile businesses want to accept flexible working futures to attract employees, and many employees are attempting to spend as little time as possible in the employer's office—and others are planning to leave employers who are averse to working from anywhere, at least for the time being. The studies collected on this Research Topic raise further questions about the suitability, impact, and sustainability of using more widespread flexible work arrangement practices. More research on the implementation and evaluation of effective flexible work arrangement practices, including but not limited to home-based teleworking, hybrid work forms, and the tools (i.e., workspace, ICT, and home office ergonomics) provided to employees and corporate leaders, is required to understand their contribution to social and economic outcomes for organizations (Eurofund, 2020). In the post-pandemic era, targeted public policies related to productivity gains from flexible work arrangement practices emanating from well-conducted studies can be beneficial to both private and public organizations (OECD, 2020). Further, communication and collaboration among social partners (employees, corporate leaders, and other stakeholders) are critical to ensuring that new, efficient, and welfare-improving work methods can be developed and maintained as standard forms of flexible work arrangement practices following the pandemic.

## Author contributions

All authors listed have made a substantial, direct, and intellectual contribution to the work and approved it for publication.
